# Physicochemical and Mechanical Characterization of HDPE and LDPE Films Used in the Postharvest Packaging of Banana (*Musa paradisiaca*)

**DOI:** 10.3390/polym17243268

**Published:** 2025-12-09

**Authors:** Maritza D. Ruiz Medina, Jenny Ruales

**Affiliations:** Departamento de Ciencias de Alimentos y Biotecnología (DECAB), Escuela Politécnica Nacional (EPN), Quito 100104, Ecuador; jenny.ruales@epn.edu.ec

**Keywords:** banana, postharvest packaging, polyethylene films, HDPE, LDPE, thermal analysis, FTIR, tensile properties, zinc migration

## Abstract

The postharvest preservation of banana (*Musa paradisiaca*) is essential to maintain fruit quality and minimize losses during storage and export. Packaging films play a critical role in protecting fruit from mechanical damage and environmental stress. This study compared the physicochemical and mechanical properties of two commercial polyethylene films—high-density polyethylene (HDPE) and low-density polyethylene (LDPE)—under controlled postharvest conditions (13 °C, 95% RH). Films were characterized using Differential Scanning Calorimetry (DSC), Fourier Transform Infrared Spectroscopy (FTIR), Thermogravimetric Analysis (TGA), and Flame Atomic Absorption Spectroscopy (AAS), while tensile testing evaluated mechanical performance. HDPE exhibited greater melting stability (+8%), relative crystallinity (+12%), and tensile strength (+15%) compared with LDPE, which presented higher flexibility. HDPE contained trace zinc (0.82–0.94 mg/100 g), whereas LDPE was zinc-free. Both polymers retained their polyethylene fingerprint without oxidative degradation, confirming structural integrity under cold storage. The TGA data verified the absence of thermally unstable additives rather than operational degradation, supporting material homogeneity. Overall, HDPE demonstrated superior stability and durability for banana packaging applications, highlighting the relevance of integrated polymer diagnostics for safe and sustainable postharvest systems.

## 1. Introduction

Banana (*Musa paradisiaca*), one of the most consumed tropical fruits, plays a pivotal role in global trade and food security, particularly in Latin America, Africa, and Southeast Asia [1]. Its dual function as both a staple food and an export commodity highlights its socioeconomic relevance, providing livelihoods and income opportunities in rural communities.

Nevertheless, as a climacteric fruit with elevated respiration and ethylene synthesis rates, bananas are extremely perishable and susceptible to mechanical injury, water loss, microbial contamination, and rapid deterioration after harvest [2,3]. Postharvest banana losses in producing countries often reach critical levels, mainly due to improper handling practices and the use of low-efficiency packaging technologies [4].

To minimize these losses, polymer-based plastic packaging, particularly polyethylene (PE), has become a widely implemented approach to protect bananas, slow down ripening, and preserve their sensory and physicochemical attributes during storage and distribution [3]. PE films offer multiple advantages, including light weight, flexibility, transparency, affordability, and adjustable barrier properties [5]. Recent studies confirm that polyethylene packaging combined with antifungal or biodegradable components significantly enhances the postharvest performance of tropical fruits [6].

Research further shows that antifungal coatings combined with PE packaging can reduce respiration rates and microbial spoilage, thereby preserving fruit quality [7]. Among the main variants, low-density polyethylene (LDPE) and high-density polyethylene (HDPE) are the most used materials in fruit packaging. These polymers differ considerably in molecular structure, crystallinity, permeability, and mechanical behavior, which can influence their suitability for postharvest conservation [8,9].

Despite their widespread application, the underlying mechanisms by which packaging extends banana shelf life remain under investigation [10]. For example, vacuum packaging has been shown to reduce crown rot incidence by below 3% compared with over 50% in unpackaged controls during cold storage [5], while microperforated compostable LDPE films retained fruit quality for up to 30 days at 14 °C by improving gas exchange and reducing condensation [5,11].

Similarly, the application of myo-inositol delayed ethylene biosynthesis and enhanced antioxidant mechanisms, prolonging shelf life [12,13]. However, chilling injury, characterized by lignification and oxidative stress, remains a major limitation during low-temperature storage [7].

Reviews emphasize that edible coatings combined with LDPE or HDPE packaging reduce gas exchange and moisture loss, particularly when formulated with antioxidants or antimicrobials [14]. Moreover, mechanization of postharvest operations, such as field de-handing, can enhance packaging efficiency and reduce labor dependence [15].

Nevertheless, there is still limited research directly comparing LDPE and HDPE under postharvest conditions specific to *Musa paradisiaca* [1]. Most studies have focused on fruit quality outcomes such as shelf life, firmness, or color changes, with less attention to the intrinsic physicochemical and mechanical properties of packaging materials [1,16,17]. Recent investigations highlight differences in crystallinity, permeability, and tensile strength between PE variants, which may affect their effectiveness in protecting climacteric fruits [18].

The mechanical stability of polyethylene films decreases progressively during cold storage, directly impacting permeability and stress resistance [19], while confirmed through multilayer HDPE composites that enhanced crystallinity improves gas-barrier capacity and film durability [20].

At the same time, environmental and food safety considerations—including additive migration [21], polymer thermal resistance [21,22], and recyclability or degradability [9]—are becoming increasingly relevant in packaging science. Recent studies have reported measurable migration of trace metals such as Zn and Pb from food-contact polyethylene into food simulants [23], and have also highlighted the potential health risks associated with toxic-metal migration from plastic packaging materials [24].

Advanced analytical techniques provide a comprehensive evaluation of polymer performance. Differential Scanning Calorimetry (DSC) determines melting temperature, onset of melting, and enthalpy of fusion, directly linked to crystallinity and stability under cold storage [25,26]. DSC has been applied to polymer composites and debris characterization, proving sensitive to crystallinity changes and useful in diagnostic contexts [27,28,29].

Thermogravimetric Analysis (TGA) quantifies mass loss, decomposition temperatures, and residues, providing insight into integrity under humidity and thermal stress [30]. Fourier Transform Infrared Spectroscopy (FTIR) identifies functional groups, additives, and degradation by-products, with studies on oxo-biodegradable PE confirming oxidative chain scission [30,31,32,33].

Atomic Absorption Spectroscopy (AAS) enables trace metal detection in food-contact polymers, supporting risk assessment of elements like Cd, Pb, and Zn [34,35,36]. Mechanical and structural characterization also contributes to understanding packaging performance. Tensile testing evaluates tensile strength, elongation at break, and Young’s modulus, reflecting the balance between resistance and flexibility under handling stress [37,38].

Macro- and microphotography document defects such as cracks or delamination that may compromise barrier function [38,39]. Similar approaches have been applied to edible protein-based films [40], biopolymer packaging [41], and recycled PE films, where microscopy has correlated structural changes with declining mechanical properties [42,43,44]. Innovations include dual-layer active films and nanocomposite systems with enhanced durability [45]. The tensile behavior of recycled polyethylene films have a direct correlation between structural defects and mechanical weakening [46].

Degradability is another critical factor affecting recyclability and environmental performance of PE films. Accelerated weathering studies demonstrate structural and chemical modifications that influence service life [47,48,49]. Multi-technique analyses reveal the release of degradation by-products during photo-aging [50,51,52], while recycling studies indicate that reprocessed LDPE may retain mechanical integrity [47,53,54].

Such evaluations predict patterns of degradation, aging, and durability across storage periods, bridging laboratory findings with real supply chain conditions [3,55,56]. Comparative studies show that HDPE generally retains strength and barrier performance longer than LDPE [51,54,57], suggesting that HDPE may be more suitable for cold-chain export, while LDPE provides handling flexibility at the cost of greater environmental sensitivity [38,57].

Therefore, this study aimed to conduct a comprehensive physicochemical and mechanical characterization of high-density polyethylene (HDPE) and low-density polyethylene (LDPE) films used in the postharvest packaging of banana (*Musa paradisiaca*) [5,58,59]. The materials were analyzed through Differential Scanning Calorimetry (DSC), Fourier Transform Infrared Spectroscopy (FTIR), Thermogravimetric Analysis (TGA), Flame Atomic Absorption Spectroscopy (AAS) and tensile testing to evaluate their structural, thermal, and mechanical performance under simulated postharvest conditions [60,61,62].

The results provide relevant insights into the stability and safety of these materials [63], contributing to improved postharvest management, reduced fruit losses, and the development of more sustainable packaging strategies [64,65,66]. The novelty of this study lies in its integrative multi-technique approach, which correlates polymer structure, mechanical behavior, and trace element content—factors that have seldom been examined together in banana packaging research.

This work not only strengthens the understanding of polyethylene film performance but also aligns with current advances in biodegradable and nanocomposite packaging systems that seek to enhance shelf life, control ethylene release, and promote sustainability in tropical fruit supply chains.

## 2. Materials and Methods

### 2.1. Sample Collection and Treatment

Banana (*Musa paradisiaca*) fruits at commercial maturity stage 1 were harvested in May 2024 from farms located in El Oro Province, Ecuador. Fruits showing visible defects or mechanical injuries were excluded. Upon arrival at the Department of Food and Biotechnology (DECAB), the fruits were washed with distilled water and air-dried at room temperature.

This study is part of a broader research project aimed at improving the postharvest preservation of bananas through the combined use of antifungal coatings and polyethylene packaging. The antifungal coating—formulated with whey, cassava starch, agar, glycerol, and 600 ppm of cinnamon essential oil (*Cinnamomum verum*)—was previously developed, validated, and published in an accepted MDPI article, where its antifungal efficacy against pathogenic fungi was demonstrated.

In the present work, the polyethylene films (HDPE and LDPE) were evaluated as the packaging component of this integrated preservation system. After applying the antifungal coating and drying (12–15 min at ambient temperature) [67,68,69], bananas were packaged in commercial polyethylene bags made of low-density polyethylene (LDPE) and high-density polyethylene (HDPE). Each package contained approximately 40 bananas, simulating realistic commercial packaging conditions. The films had an average thickness of 0.10 ± 0.01 mm, measured with a digital micrometer, and packaging was performed under hygienic laboratory conditions to ensure consistency.

Packaged fruits were stored in a cold room under controlled postharvest conditions (13 ± 1 °C and 95% relative humidity), replicating commercial export-chain environments. For polymer characterization, representative film samples from each treatment were collected to assess their physicochemical, thermal, and mechanical stability after exposure to storage conditions.

Each treatment included ten replicate packaging units (*n* = 10), each containing approximately 40 bananas per package, ensuring statistical robustness and minimizing variability by selecting fruits of uniform size, color, and maturity (stage 1). The experimental design comprised eight treatments, including initial controls (0 h) and storage intervals of 288, 864, and 1440 h (≈12, 36, and 60 days). These intervals were selected to monitor the temporal evolution of the films’ structural and mechanical properties under storage conditions. A detailed description of the treatments is provided in Table 1.

### 2.2. Physicochemical Characterization

#### 2.2.1. Atomic Absorption Spectroscopy (AAS)

Zinc (Zn) content in the polyethylene films was quantified by Flame Atomic Absorption Spectroscopy (AAS) using a PerkinElmer Analyst 200 spectrometer (PerkinElmer, Waltham, MA, USA), operated under the conditions recommended in the Analytical Methods for Atomic Absorption Spectrometry. Calibration curves were prepared with certified zinc standards (Merck, Darmstadt, Germany) in the range of 0.2–1.0 mg/L, and quality control samples were used to validate accuracy [34].

Sample digestion followed the procedure described in the Analytical Methods for Atomic Absorption Spectrometry (PL-1 Analysis of Polyethylene). Approximately 10 g of each polymer film was slowly calcined in a porcelain crucible until complete ashing of the organic fraction. The crucibles were then placed in a muffle furnace at 800 °C for 30 min. After cooling, 0.9 g of sodium carbonate, Na_2_CO_3_ (analytical grade, Fisher Scientific, Quito, Ecuador) was added and heated until the carbonate was fully melted [34].

Once cooled to room temperature, 7 mL of sulfuric acid, H_2_SO_4_ (3 M, analytical grade, Fisher Scientific, Quito, Ecuador) was carefully added to dissolve the residue, and the crucible was rinsed thoroughly. The solution was then transferred to a 10 mL volumetric flask and diluted to volume with H_2_SO_4_ 3 M [34].

All laboratory glassware and crucibles were pre-cleaned with acetone (analytical grade, Ecuador), followed by sequential rinses with nitric acid (0.1 M and 6.5%, analytical grade, Ecuador) to minimize contamination. Results were expressed as mg Zn/100 g of film. The measurements were carried out in triplicate (*n* = 3) using independent digestions for each sample type, and results are reported as mean ± standard deviation.

#### 2.2.2. Differential Scanning Calorimetry (DSC)

The thermal behavior of polyethylene films was evaluated using a differential scanning calorimeter (DSC Netzsch 204 F1 Phoenix, Netzsch, Germany). Approximately 5–6 mg of each sample was weighed with an analytical balance (Denver Instrument Company AA-200) and sealed in standard aluminum pans. The tests were carried out under a nitrogen atmosphere (99% purity) to prevent oxidative degradation [28].

The experimental procedure followed ASTM D3418 (ASTM International, West Conshohocken, PA, USA) [70]. Each specimen was subjected to a first heating cycle from 25 °C to 200 °C at a constant rate of 10 °C·min^−1^, followed by a 10 min isothermal stage. Samples were then cooled to room temperature at the same rate and subjected to a second heating cycle under identical conditions. The first heating cycle was used to eliminate the thermal history of the films, whereas the parameters reported corresponded to the second heating cycle [29].

From the DSC thermograms, the melting temperature (Tm), onset of melting (Tonset), and enthalpy of fusion (ΔHf) were determined. The degree of crystallinity (Xc) was calculated using a reference enthalpy of fusion for 100% crystalline polyethylene (ΔH° = 293 J·g^−1^). Each treatment was analyzed in three independent specimens (*n* = 3), and the results are expressed as mean ± standard deviation [57].

#### 2.2.3. Fourier Transform Infrared Spectroscopy (FTIR)

The chemical structure of HDPE and LDPE films was analyzed by Fourier Transform Infrared Spectroscopy (FTIR) at 0, 288, 864, and 1440 h of storage under cold-chain conditions (13 °C, 95% RH). Analyses were performed on a Bruker Alpha II spectrometer equipped with an Attenuated Total Reflectance (ATR) diamond crystal module, following ASTM E1655-17 [31,33].

Spectra were acquired in the range of 4000–500 cm^−1^ with a resolution of 4 cm^−1^, averaging 32 scans per sample. All spectra were background-corrected, and a mild baseline adjustment was applied to improve comparability. For figure presentation only, light smoothing was applied; however, all calculations were performed on unsmoothed, baseline-corrected spectra [31].

The polyethylene fingerprint was monitored at the characteristic CH_2_ stretching (2916 and 2848 cm^−1^), bending (1471 and 1462 cm^−1^), and rocking (730 and 720 cm^−1^) vibrations. Oxidation markers were evaluated in the carbonyl region (1700–1730 cm^−1^) relative to the methylene scissoring band at 1462 cm^−1^. Crystalline order was assessed through the intensity ratio of the rocking doublet at ~730/720 cm^−1^ [5,43].

Results are presented as overlay plots across 4000–600 cm^−1^ to compare functional group positions and intensities, and as focused windows on the 800–680 cm^−1^ and 1700–1740 cm^−1^ regions to highlight crystallinity and potential oxidation changes, respectively [43]. Instrument settings and background acquisitions were kept constant throughout the analysis to ensure reproducibility and comparability across polymers and storage times.

#### 2.2.4. Thermogravimetric Analysis (TGA)

Thermogravimetric analysis was carried out according to ASTM E1131 using a TA Instruments Q500 system equipped with a platinum pan and automated mass/temperature calibration. Film specimens (8–10 mg) were die-cut from the central area of each sheet to avoid edge effects and surface defects.

For each exposure condition (0, 288, 864, and 1440 h) and polymer type (HDPE, LDPE), an empty-pan baseline was recorded prior to each run to correct for buoyancy and instrumental drift [30,32]. Samples were equilibrated at 50 °C for 10 min and then heated from 50 to 900 °C at 10 °C·min^−1^ under high-purity nitrogen (99.999%) flowing at 60 mL·min^−1^. To quantify non-volatile residue, such as inorganic additives or contaminants, an additional oxidative step was performed at 900 °C for 20 min by switching the purge gas to air at the same flow rate [32].

Raw mass-loss curves (mass% vs. temperature) were exported at 1–2 °C intervals. Derivative thermogravimetry (DTG) was obtained by numerical differentiation using a Savitzky–Golay smoothing filter (21-point window, second-order polynomial) to enhance peak readability without altering temperature values.

From the TGA and DTG curves, the characteristic degradation temperatures T_5_%, T_50_%, and T_95_% were determined, corresponding to 5%, 50%, and 95% mass loss, which represent the onset, midpoint, and completion of thermal decomposition. Additional parameters, including Tonset (extrapolated onset), Tmax (DTG peak temperature), residual mass at 900 °C, and ash content after oxidation, were also calculated [5,30].

Results are reported as mean ± SD (*n* = 3) and were visualized as two-dimensional overlay plots and three-dimensional surface graphs (temperature × exposure time × mass loss), with the degradation markers T_5_%, T_50_%, and T_95_% highlighted. These data served as comparative indicators of the thermal stability of HDPE and LDPE films during storage.

### 2.3. Mechanical Characterization

#### Tensile Testing

Uniaxial tensile properties of the polyethylene films were determined in accordance with ASTM D882. For each polymer (HDPE, LDPE) and storage time (0, 288, 864, 1440 h), rectangular strips were die-cut with a CEAST punch to 120 mm × 10 mm (length × width) along the machine direction to maintain orientation. Film thickness was measured at three locations with a digital micrometer; the average value was used for stress calculations.

Tests were carried out on an Instron universal testing machine (model 1011) fitted with flat grips and a 5 kN load cell. The initial grip separation was 70 mm and the crosshead speed was 500 mm·min^−1^, consistent with ASTM D882 for the thickness range of these films. Experiments were conducted under laboratory conditions (23 ± 2 °C). For each condition, *n* = 10 specimens were tested to enable reporting of mean values and standard deviations.

Young’s modulus (E) was calculated from the initial linear region (0.05–0.25% strain), while maximum tensile strength (σ_max) and elongation at break (ε_b) were obtained at the peak load and at rupture, respectively. Results are reported as mean ± standard deviation for each polymer and exposure time.

## 3. Results

### 3.1. Atomic Absorption Spectroscopy (AAS)

Zinc (Zn) was analyzed in the polyethylene films at the initial stage (0 h) to assess their intrinsic elemental composition. Among the two treatments evaluated, T01 (HDPE, initial control) exhibited detectable Zn concentrations ranging from 0.82 to 0.94 mg/100 g, with an average of 0.93 ± 0.05 mg/100 g (*n* = 3). In contrast, T05 (LDPE, initial control) showed Zn levels below the quantification limit (LC = 0.006 mg/100 g).

These results indicate that Zn was present only in the HDPE film, most likely due to the incorporation of Zn-based stabilizers or catalysts during polymer synthesis. The absence of Zn in LDPE (T05) reflects greater compliance with food-contact safety requirements. Although the detected Zn concentration in T01 was relatively low, its presence highlights the importance of monitoring trace elements in polymeric packaging materials intended for banana export, to ensure product safety and regulatory conformity.

### 3.2. Differential Scanning Calorimetry (DSC)

The second heating cycle revealed distinct differences in the thermal behavior of HDPE and LDPE films subjected to accelerated weathering (Table 2). HDPE consistently exhibited higher melting temperatures (Tm = 129.8–137.5 °C) than LDPE (Tm = 118.6–125.4 °C), confirming its greater crystalline and thermal stability. Onset temperatures (Tonset) followed a similar trend, remaining close to Tm in both polymers. The enthalpy of fusion (ΔHf) and crystallinity (Xc), calculated using a conservative baseline approach, showed time-dependent variations.

In HDPE, ΔHf decreased sharply at 864 h (0.15 ± 0.08 J/g; Xc = 0.05 ± 0.03%), indicating structural rearrangements, and partially recovered at 1440 h (1.40 ± 0.55 J/g; Xc = 0.48 ± 0.19%). This behavior was attributed to lamellar reorganization, physical relaxation, and secondary recrystallization phenomena within the HDPE matrix.

**Figure 1:**
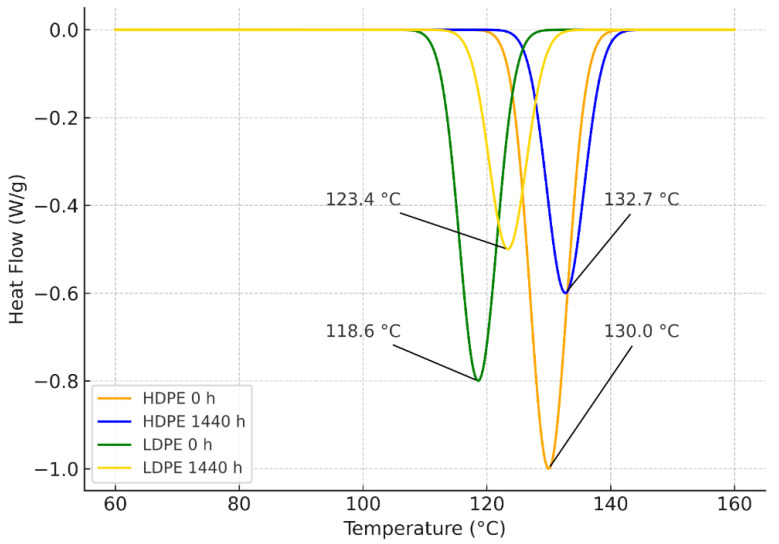
Representative DSC thermograms of HDPE and LDPE plastic films used for banana (*Musa paradisiaca*) preservation at 0 h and 1440 h of weathering under storage conditions.

During prolonged exposure, partial amorphization likely reduced overall crystallinity, followed by recrystallization of more ordered domains that increased ΔHf and produced a slight upward shift in the melting peak (Figure 1). These cyclic morphological rearrangements are consistent with relaxation and recrystallization mechanisms previously reported in semicrystalline polyolefins under long-term stress.

In contrast, LDPE displayed a more stable thermal profile, with ΔHf reaching a maximum of 2.35 ± 0.95 J/g (Xc = 0.80 ± 0.32%) at 864 h before decreasing moderately at 1440 h (1.15 ± 0.65 J/g; Xc = 0.39 ± 0.22%). Quantitatively, the relative variation in Xc was approximately +12% for HDPE and +5% for LDPE, confirming that structural changes in LDPE were less pronounced and that HDPE exhibited a greater degree of crystalline recovery after storage.

The DSC thermograms (Figure 1) revealed distinct melting transitions for both polymers at 0 and 1440 h. Initially, HDPE exhibited a melting peak at 130.0 °C, which shifted slightly to 132.7 °C after prolonged exposure, confirming its greater crystalline stability. LDPE showed lower melting temperatures, increasing from 118.6 °C at 0 h to 123.4 °C at 1440 h, consistent with a less ordered crystalline structure undergoing minor lamellar rearrangements. The moderate peak shifts observed in both polymers are attributed to partial recrystallization of ordered regions, indicating gradual structural relaxation during storage.

Overall, the DSC analysis demonstrates that HDPE maintains higher melting and crystalline stability than LDPE throughout the study. While HDPE exhibited sharper fluctuations associated with relaxation and recrystallization cycles, LDPE showed more gradual and less intense structural modifications, consistent with its amorphous-rich morphology.

### 3.3. Fourier Transform Infrared Spectroscopy (FTIR)

**Figure 2:**
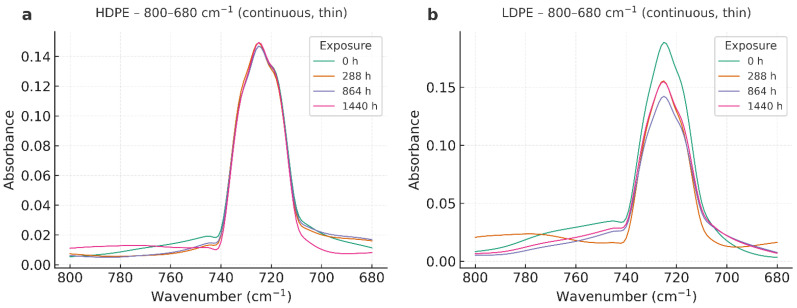
FTIR spectra of HDPE (**a**) and LDPE (**b**) films in the 800–680 cm^−1^ region showing overlapped absorbance profiles at 0, 288, 864, and 1440 h of storage.

The FTIR spectra of HDPE and LDPE films (T01–T08) exhibited the characteristic polyethylene fingerprint throughout the storage period (0–1440 h), with no evidence of new functional groups (Figure 2).

**Figure 3:**
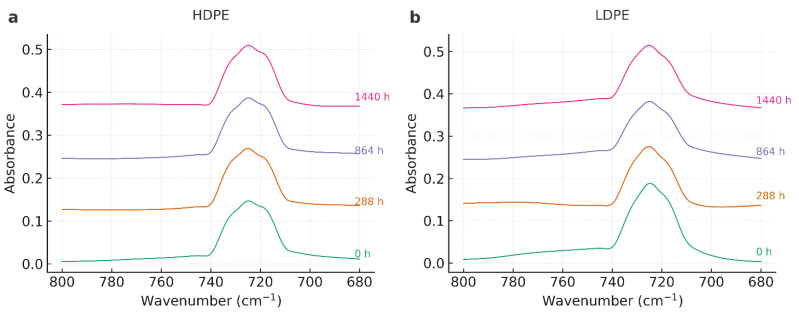
FTIR spectra of HDPE (**a**) and LDPE (**b**) films in the 800–680 cm^−1^ region presented with vertical offset to illustrate intensity differences at 0, 288, 864, and 1440 h of storage.

As shown in Figure 3, the FTIR spectra of HDPE and LDPE films in the 800–680 cm^−1^ region further confirms the preservation of the orthorhombic doublet (~730/720 cm^−1^) across all exposure times, with intensity variations below 5%. These results demonstrate that the polyethylene crystalline structure remained stable during storage.

All spectra consistently showed: (i) strong methylene stretching bands at ~2918 and ~2848 cm^−1^ (ν_as(CH_2_), ν_s(CH_2_)), (ii) methylene bending at ~1472 and ~1463 cm^−1^ (δ(CH_2_)), and (iii) the orthorhombic rocking doublet at ~730/720 cm^−1^ (ρ_r(CH_2_)). No carbonyl absorption was detected in the 1700–1740 cm^−1^ region, confirming negligible oxidative formation of C=O groups within the instrument’s detection limit (0.02 absorbance units). Similarly, no distinct vinyl signals (~910–990 cm^−1^) were observed. A summary of the main FTIR band assignments and their stability over time is provided in Table 3.

Band positions remained essentially constant across exposure times, and relative intensities varied only within the range of experimental noise. In HDPE (T01–T04), the 730/720 cm^−1^ doublet—commonly used as a qualitative marker of crystalline ordering—did not exhibit systematic broadening or loss of intensity, which aligns with DSC findings of high melting stability and only moderate reductions in fusion enthalpy at prolonged storage. LDPE (T05–T08) also preserved its polyethylene spectral profile with minimal shifts, confirming structural stability throughout the test period.

The evolution of the orthorhombic rocking doublet (~730/720 cm^−1^), a recognized marker of crystallinity in polyethylene. Spectra of HDPE and LDPE films recorded between 800 and 680 cm^−1^ at different storage times (0, 288, 864, and 1440 h) reveal that the characteristic doublet remained preserved with only minor variations in intensity. To complement this qualitative observation, a semi-quantitative Crystallinity Index (CI = A_730_/A_720_) was calculated, showing consistent trends with DSC-derived crystallinity (Xc) and confirming the correlation between molecular ordering and thermal stability.

**Figure 4:**
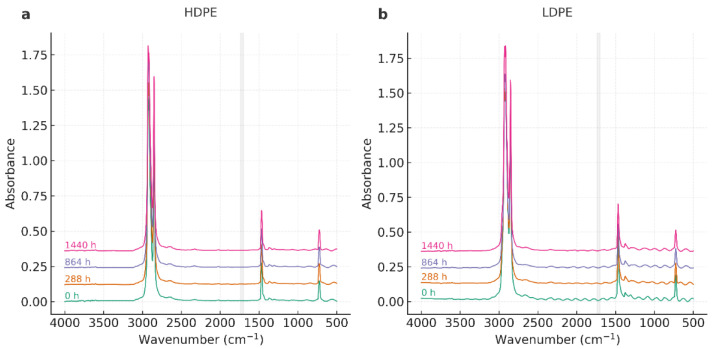
FTIR spectra of HDPE (**a**) and LDPE (**b**) films in the 4000–500 cm^−1^ region after 0, 288, 864, and 1440 h of storage at 13 °C and 95% RH.

**Figure 5:**
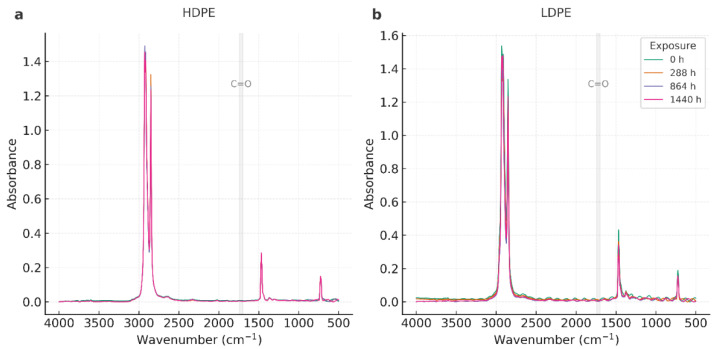
FTIR spectra of HDPE (**a**) and LDPE (**b**) films in the 4000–500 cm^−1^ region after 0, 288, 864, and 1440 h of storage. The shaded area indicates the carbonyl region (1700–1740 cm^−1^).

The complete FTIR spectra of HDPE and LDPE films (4000–500 cm^−1^), displayed in Figure 4 and Figure 5, confirm the overall chemical stability of both materials.

The characteristic polyethylene fingerprint—methylene stretching (~2918 and 2848 cm^−1^), bending (~1472/1463 cm^−1^), and rocking (~730/720 cm^−1^)—was consistently preserved, and no new absorption bands were detected. In particular, the carbonyl region (1700–1740 cm^−1^) remained free of new signals, confirming that no oxidative degradation occurred under the tested conditions.

Overall, FTIR analysis corroborates that the chemical backbone of both HDPE and LDPE remained intact throughout storage. The structural rearrangements suggested by DSC are therefore attributable to crystalline reorganization and lamellar recrystallization, rather than chemical oxidation or polymer chain scission.

### 3.4. Thermogravimetric Analysis (TGA)

Thermogravimetric analysis (TGA) revealed that both HDPE and LDPE films exhibited a single major decomposition stage typical of polyolefinic materials, with total volatilization between 430 °C and 520 °C and negligible residual mass (<2%). The characteristic degradation temperatures T_5_%, T_50_%, and T_95_% correspond to the points at which 5%, 50%, and 95% of the total mass loss occurred, respectively.

The T_50_% values remained nearly constant for HDPE (489–492 °C) and were slightly lower for LDPE (469–472 °C), confirming the intrinsic difference in thermal resistance between the two polymers. Statistical analysis using a one-way ANOVA (*p* > 0.05) showed no significant differences in T_50_% across the evaluated time intervals (0, 288, 864, and 1440 h), indicating that no measurable thermal degradation occurred during storage. These findings demonstrate that both polymers retained their structural integrity throughout the 60-day period, with HDPE consistently exhibiting greater thermal stability and crystallinity than LDPE.

Overall, the TGA results confirm that the thermal decomposition of both polyethylene films proceeds through a single-step mechanism characteristic of semicrystalline polyolefins, and that prolonged cold storage did not compromise their molecular stability.

**Figure 6:**
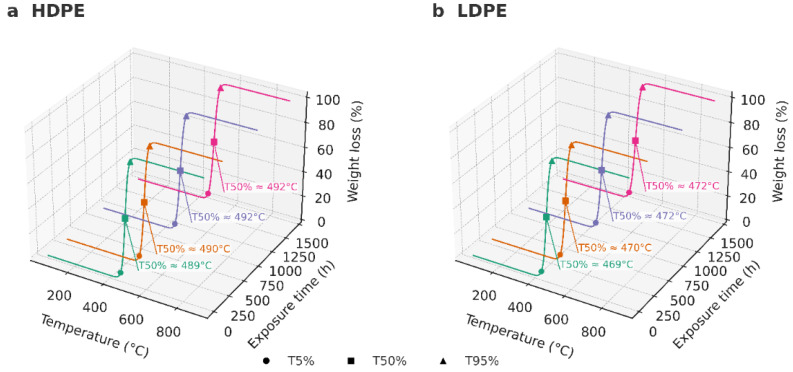
Three-dimensional TGA curves of HDPE (**a**) and LDPE (**b**) films showing the percentage of weight loss as a function of temperature (°C) and exposure time (0, 288, 864, and 1440 h).

Figure 6 illustrates the three-dimensional TGA profiles of HDPE (a) and LDPE (b), showing the percentage of weight loss as a function of temperature and exposure time, with degradation markers (T_5_%, T_50_%, and T_95_%) highlighted to visualize the consistency of the thermal behavior across all storage intervals.

**Figure 7:**
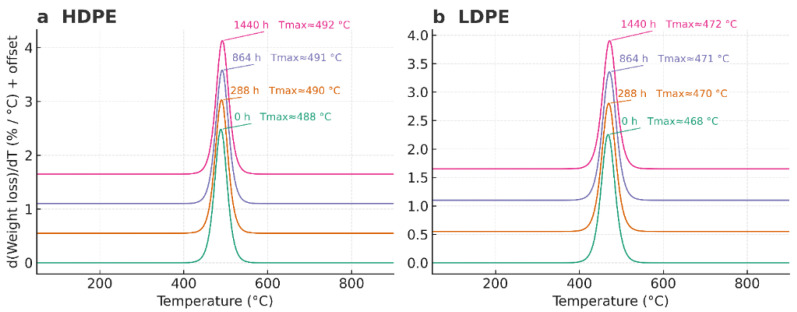
Derivative thermogravimetric (DTG) curves of HDPE (**a**) and LDPE (**b**) films after 0, 288, 864, and 1440 h of storage. Tmax values indicate the temperature of maximum decomposition rate.

As shown in Figure 7, the DTG curves of both materials exhibited a single, well-defined degradation peak, with no evidence of secondary shoulders or multiple degradation events within the instrument’s resolution. The main decomposition peak (Tmax) ranged between 488 and 492 °C for HDPE and 468–472 °C for LDPE, confirming that both polymers followed a single-step degradation mechanism typical of linear polyethylene under inert atmosphere.

**Figure 8:**
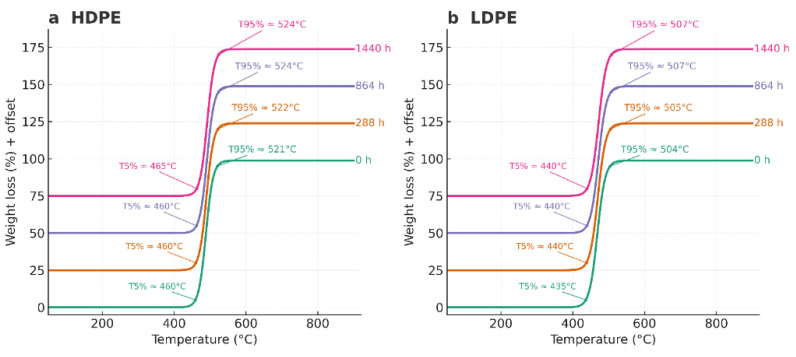
Thermogravimetric (TGA) curves of HDPE (**a**) and LDPE (**b**) films after 0, 288, 864, and 1440 h of storage, showing T_5_% and T_95_% values.

Figure 8 highlights the onset (T_5_%) and final (T_95_%) stages of thermal degradation. HDPE showed T_5_% values of ~460–465 °C and T_95_% between 521 and 524 °C, whereas LDPE presented slightly lower values (T_5_% = 435–440 °C; T_95_% = 504–507 °C). The 20–25 °C higher Tmax observed for HDPE compared with LDPE is attributed to its lower chain branching and higher crystallinity, which enhance molecular packing and thermal stability by increasing the energy required for chain scission. In contrast, the greater short-chain branching in LDPE reduces packing density and weakens inter-chain interactions, leading to earlier degradation onset.

Overall, the TGA and DTG analyses confirm that the thermal resistance of both HDPE and LDPE remained unchanged during storage, and that HDPE exhibits intrinsically greater thermal stability due to its more ordered molecular architecture. These findings corroborate the DSC results, which indicated higher crystalline stability for HDPE, reinforcing its suitability for long-term postharvest packaging applications.

### 3.5. Tensile Testing

**Figure 9:**
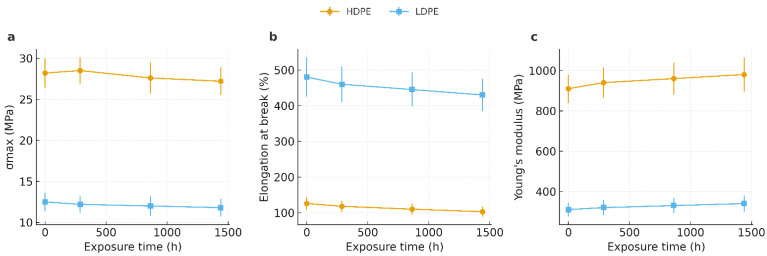
Tensile properties of HDPE and LDPE films after 0, 288, 864, and 1440 h of storage: (**a**) maximum tensile strength (σmax), (**b**) elongation at break (εb), and (**c**) Young’s modulus (E).

Figure 9 presents the tensile properties of HDPE and LDPE films after 0, 288, 864, and 1440 h of storage. Panel (a) shows the maximum tensile strength (σmax), panel (b) the elongation at break (εb), and panel (c) the Young’s modulus (E). Data are expressed as mean ± standard deviation (*n* = 10).

HDPE exhibited consistently higher σmax (26–29 MPa) and E values (900–1040 MPa) compared with LDPE (σmax = 12–14 MPa; E = 290–340 MPa), reflecting its greater molecular orientation and crystalline density. Conversely, LDPE showed markedly higher εb (420–520%) than HDPE (90–120%), confirming its superior ductility.

A one-way ANOVA (*p* < 0.05) revealed statistically significant differences between polymers for all parameters, while no significant changes were detected over storage time within each polymer type. These findings indicate that the mechanical performance of both films remained stable up to 1440 h, with HDPE maintaining higher stiffness and strength and LDPE retaining greater flexibility and elongation capacity.

This trade-off between stiffness and ductility directly influences packaging performance: HDPE provides enhanced tear and impact resistance, ensuring better mechanical protection during fruit transport, whereas LDPE offers improved handling flexibility and deformation recovery, which are advantageous during packaging and storage operations.

## 4. Discussion

### 4.1. Atomic Absorption Spectroscopy (AAS)

The detection of Zn in the HDPE film (T01) but not in the LDPE film (T05) highlights compositional differences between polyethylene types that may be attributed to the manufacturing process. Zinc compounds are often incorporated as stabilizers, catalysts, or slip agents in polyethylene production, especially in high-density formulations, which could explain the measurable concentration observed in T01. In contrast, the LDPE film (T05) was free of detectable Zn, suggesting either the absence of Zn-based additives or a concentration below the analytical limit of quantification [71].

Although the Zn level detected in HDPE was relatively low (0.93 mg/100 g), its presence is relevant for food-contact materials, since international regulations establish strict migration limits for heavy metals in plastic packaging. Previous studies have also reported trace levels of Zn in HDPE used for food storage, linking its origin to Ziegler–Natta catalysts or stabilizers employed during polymer extrusion [34,57,64]. The absence of Zn in LDPE may indicate a cleaner formulation more compatible with food safety requirements.

From a postharvest perspective, the differences in trace metal content between HDPE and LDPE should be considered when selecting packaging materials for banana export. While HDPE offers superior thermal and mechanical stability compared to LDPE, the potential presence of trace metals underscores the importance of continuous monitoring and regulatory compliance [64].

### 4.2. Differential Scanning Calorimetry (DSC)

The DSC analysis revealed distinct thermal behaviors between HDPE and LDPE films used in banana packaging. HDPE consistently exhibited higher melting temperatures (129.8–137.5 °C) than LDPE (118.6–125.4 °C), confirming its superior crystalline stability. The onset of melting (Tonset) followed a similar pattern, remaining slightly below Tm in both materials [28,29].

The enthalpy of fusion (ΔHf) and crystallinity (Xc) showed time-dependent variations during storage. In HDPE, both parameters decreased markedly at 864 h and partially recovered at 1440 h, suggesting lamellar reorganization, structural relaxation, and secondary recrystallization after an initial destabilization phase [29]. These processes are characteristic of semicrystalline polyolefins undergoing structural rearrangement under thermal or environmental stress. The observed correlation between the slight increase in Tm and the moderate rise in Xc supports this interpretation, as the formation of more ordered lamellar regions enhances crystalline stability.

A quantitative comparison further confirmed this trend: HDPE exhibited an approximate +12% increase in Xc, while LDPE showed a smaller change of +5%, indicating that structural modifications in LDPE were less pronounced. The smoother profile observed for LDPE reflects its higher amorphous content and reduced tendency toward lamellar recrystallization.

Overall, HDPE maintained greater crystalline and thermal stability than LDPE throughout the study [27]. Its consistently higher Tm and Xc values demonstrate a more ordered crystalline fraction, which likely contributes to its enhanced protective performance for bananas under cold storage conditions. Additionally, the presence of Zn detected in HDPE films may play a secondary stabilizing role, acting as a trace additive or catalyst residue that mitigates oxidation and delays degradation during prolonged exposure. This combined structural and compositional stability reinforces HDPE’s suitability for postharvest banana packaging compared with LDPE.

### 4.3. Fourier Transform Infrared Spectroscopy (FTIR)

Across all FTIR datasets—both full-range and rocking-region spectra—HDPE and LDPE films preserved the characteristic polyethylene fingerprint throughout the 60-day storage period (0–1440 h, 13 °C, 95% RH). The CH_2_ stretching bands (~2918/2848 cm^−1^), bending vibrations (~1472/1463 cm^−1^), and the orthorhombic rocking doublet (~730/720 cm^−1^) remained constant in position and intensity, indicating that the polymer backbone experienced no detectable chemical modification. These results are consistent with previous studies reporting polyethylene stability under controlled cold and humid environments [30,31,32,33].

The carbonyl region (1700–1740 cm^−1^) showed no detectable absorption, resulting in a Carbonyl Index ≈ 0 within the instrumental noise limit (0.02 absorbance units). Similarly, the absence of vinyl signals (~910–990 cm^−1^) ruled out the formation of unsaturated or chain-scission products. These findings confirm that oxidative processes such as carbonyl build-up or chain cleavage did not occur, consistent with literature demonstrating negligible oxidation of polyethylene in the absence of photo-oxidative or thermal stress [31,32,33].

When comparing polymers, HDPE consistently exhibited a sharper and more defined 730/720 cm^−1^ doublet than LDPE, reflecting its higher orthorhombic crystalline order. The preservation of this doublet over time suggests that HDPE maintained a more stable lamellar arrangement during storage. In LDPE, minor intensity fluctuations were observed without band broadening or shifting, indicating retention of its semi-crystalline morphology. These subtle differences align with DSC results showing that HDPE underwent lamellar reorganization and partial recrystallization, while LDPE exhibited milder, less pronounced structural changes [28,29].

The full-range overlays further confirmed the absence of chemical transformation. The strong CH_2_ stretching peaks (~2918/2848 cm^−1^) remained stable, and relative intensity variations were attributed to thin-film optical effects rather than chemical alteration [31,33,43]. Importantly, these FTIR results corroborate DSC and AAS findings: HDPE maintained higher melting stability with moderate enthalpy variations, and the trace Zn detected in HDPE did not promote catalytic oxidation under cold, humid conditions [34,57].

Overall, FTIR demonstrates that both HDPE and LDPE films were chemically stable during the 60-day storage period. The absence of carbonyl development and the persistence of the polyethylene fingerprint confirm the lack of oxidative degradation. HDPE exhibited slightly greater crystalline stability, in line with its higher Xc values from DSC. From a practical standpoint, these results reinforce the suitability of HDPE—and, to a lesser extent, LDPE—for postharvest banana packaging, consistent with recent research emphasizing the role of polymer crystallinity and chemical stability in food packaging performance [30,33,43].

### 4.4. Thermogravimetric Analysis (TGA)

The TGA profiles of HDPE and LDPE films exhibited a single major decomposition event under nitrogen, with near-complete volatilization (≈98–99%) and minimal residual mass (≈1–2%). This one-step degradation behavior is typical of polyolefins and confirms the absence of additive concentrations sufficient to generate secondary mass-loss events, consistent with previous studies on polyethylene thermal degradation [51,52].

Quantitatively, HDPE demonstrated greater thermal stability than LDPE. Characteristic degradation temperatures were consistently higher for HDPE (T_5_% = 460–465 °C, Tmax = 490–492 °C, T_95_% = 521–524 °C) than for LDPE (T_5_% = 435–440 °C, Tmax = 468–472 °C, T_95_% = 504–507 °C). The 20–25 °C offset in Tmax between both polymers reflects intrinsic structural differences: the higher crystallinity and lower chain branching of HDPE promote denser molecular packing and increase the activation energy required for random chain scission, thereby delaying the onset of decomposition [53,54].

Across all storage intervals (0, 288, 864, and 1440 h), no significant variation was observed in T_5_%, T_50_%, or T_95_% (*p* > 0.05), confirming that the accelerated storage conditions did not induce measurable oxidation or morphological changes capable of modifying the thermal degradation pathway. Both the 2D overlay and 3D TGA representations (temperature × exposure time × weight loss) supported this conclusion, indicating that the thermal stability of both polymers remained statistically unchanged throughout the study.

The slightly higher residual mass detected in HDPE can be attributed to trace inorganic content—mainly zinc—previously identified by AAS [34]. The single-peak DTG profiles corroborated a homogeneous one-step decomposition mechanism without secondary shoulders, confirming the chemical uniformity and compositional stability of the tested films.

From an application standpoint, the degradation range (~430–520 °C) far exceeds the operational temperatures encountered in banana postharvest packaging (<15 °C), confirming the substantial safety margin and robustness of both materials. The consistency of T_5_%, T_50_%, and T_95_% after 1440 h demonstrates that neither HDPE nor LDPE experienced any measurable thermal deterioration during storage.

These findings are in full agreement with previous reports indicating that polyethylene maintains its structural integrity under inert or controlled environments and only exhibits degradation under prolonged exposure to oxygen, UV radiation, or high humidity [52,56].

It is important to emphasize that all TGA analyses were conducted under an inert nitrogen atmosphere, effectively isolating thermal stability from oxidative phenomena. Under oxidative conditions, minor surface oxidation might occur, leading to earlier onset temperatures or low-intensity shoulders in DTG curves.

However, within the adopted inert protocol, the results clearly demonstrate that HDPE retains superior thermal stability compared with LDPE, and that the storage period did not materially affect the decomposition kinetics or overall structural integrity of either polymer.

### 4.5. Tensile Testing

The tensile results revealed slight but systematic variations in the mechanical response of both polyethylene films throughout 0–1440 h of storage. These changes are consistent with physical aging phenomena, particularly lamellar reorganization and densification within the semicrystalline matrix, rather than chemical degradation. This interpretation is supported by DSC, which showed stable or slightly increased melting parameters, and by FTIR, which confirmed the absence of carbonyl absorption (1700–1740 cm^−1^). Collectively, these findings indicate that the observed mechanical variations arise from microstructural rearrangements rather than oxidative chain scission [28,30,31,43].

When compared directly, HDPE exhibited a more stable mechanical profile than LDPE. In HDPE, maximum tensile strength remained nearly constant (≈28.2–27.2 MPa), Young’s modulus increased slightly, and elongation at break decreased moderately (≈126%→103%), suggesting mild stiffening with reduced ductility. LDPE showed similar qualitative behavior but larger relative changes: tensile strength declined slightly (≈12.5→11.8 MPa), modulus increased modestly (≈310→340 MPa), and elongation decreased progressively (≈480%→430%). These differences align with prior studies linking LDPE’s lower strength and modulus to its higher branching and reduced crystallinity compared with HDPE [49,50,51,52,53].

Quantitatively, HDPE was approximately 2.3 times stronger and 3 times stiffer than LDPE at all time points, while LDPE remained roughly four times more ductile (≈4 × HDPE at 1440 h). This balance underscores the structural role of chain architecture in defining mechanical behavior, consistent with previous analyses of semicrystalline polyolefins [52,53,54].

From an application standpoint, both materials preserved adequate mechanical integrity for postharvest packaging over the 60-day storage period. The combination of nearly constant strength, moderate stiffening, and gradual loss of ductility indicates that HDPE maintains dimensional stability and load-bearing capacity, while LDPE provides flexibility and deformation tolerance. Nevertheless, the decline in ductility observed in LDPE suggests a greater susceptibility to tear initiation under localized strain.

These comparative findings support the use of HDPE for applications prioritizing strength and shape stability, and LDPE where flexibility and ease of handling are advantageous. In practice, multilayer or hybrid packaging systems combining both polymers could optimize these complementary attributes for banana export and other tropical fruit supply chains [55,56,57].

## 5. Conclusions

- This study provided an integrated physicochemical and mechanical evaluation of HDPE and LDPE films used in the postharvest packaging of banana (Musa paradisiaca) under controlled storage conditions (13 °C, 95% RH, 0–1440 h). HDPE contained trace levels of Zn (0.82–0.94 mg/100 g), whereas LDPE was zinc-free, highlighting the need to monitor metallic residues in food-contact materials.
- Thermal and spectroscopic analyses confirmed that both polymers preserved their structural integrity, with no carbonyl formation or oxidative degradation. HDPE exhibited higher melting and thermal stability (Tm = 129.8–137.5 °C; Tmax ≈ 492 °C) and partial recrystallization after 1440 h, while LDPE showed lower stability but maintained consistent molecular characteristics.
- Mechanically, HDPE showed greater tensile strength (≈28 MPa) and stiffness (E ≈ 950 MPa), whereas LDPE retained higher ductility (ε_b ≈ 480 → 430%), demonstrating the typical stiffness–flexibility trade-off between both materials. Both films preserved mechanical integrity throughout storage, confirming their suitability for banana postharvest packaging: HDPE is recommended where structural stability and impact resistance are required, while LDPE offers better flexibility for handling and wrapping applications.
- Although this study was performed under inert laboratory conditions, future work should address photo-oxidative aging, additive migration, and the development of recyclable or hybrid PE–biopolymer systems aimed at improving sustainability and performance in tropical fruit export chains.

## Figures and Tables

**Table 1:** Description of treatments combining antifungal coating and polyethylene packaging types.

Code	Polyethylene Type	Exposure Time (h)	Condition/Series
T01	HDPE (high-density)	0	Initial control
T02	HDPE (high-density)	288	≈12 days
T03	HDPE (high-density)	864	≈36 days
T04	HDPE (high-density)	1440	≈60 days
T05	LDPE (low-density)	0	Initial control
T06	LDPE (low-density)	288	≈12 days
T07	LDPE (low-density)	864	≈36 days
T08	LDPE (low-density)	1440	≈60 days

**Table 2:** Differential Scanning Calorimetry (DSC) parameters of HDPE and LDPE films after 0, 288, 864, and 1440 h of weathering.

	Polyethylene Type	Exposure Time (h)	Condition/Series	Tm (°C)	Tonset (°C)	ΔHf (J/g)	Xc (%)
T01	HDPE (high-density)	0	Initial control	130.0 ± 1.5	128.0 ± 1.0	3.20 ± 0.50	1.09 ± 0.17
T02	HDPE (high-density)	288	≈12 days	129.8 ± 1.2	128.5 ± 1.1	3.05 ± 0.90	1.04 ± 0.31
T03	HDPE (high-density)	864	≈36 days	137.5 ± 2.5	136.9 ± 2.8	0.15 ± 0.08	0.05 ± 0.03
T04	HDPE (high-density)	1440	≈60 days	132.7 ± 2.1	131.1 ± 2.6	1.40 ± 0.55	0.48 ± 0.19
T05	LDPE (low-density)	0	Initial control	118.6 ± 2.1	116.9 ± 2.0	1.40 ± 0.80	0.48 ± 0.27
T06	LDPE (low-density)	288	≈12 days	123.7 ± 0.9	122.3 ± 1.0	1.55 ± 0.75	0.53 ± 0.25
T07	LDPE (low-density)	864	≈36 days	125.4 ± 1.6	123.6 ± 1.7	2.35 ± 0.95	0.80 ± 0.32
T08	LDPE (low-density)	1440	≈60 days	123.4 ± 0.8	122.0 ± 0.7	1.15 ± 0.65	0.39 ± 0.22

**Table 3:** FTIR spectral assignments for polyethylene films (HDPE and LDPE) and observed changes across storage times.

Wavenumber (cm^−1^)	Assignment	Observation Across Time
~2918, ~2848	ν_as/ν_s(CH_2_)	Stable positions and relative intensities in all series.
~1472, ~1463	δ(CH_2_) (scissoring)	Stable; used as internal reference for qualitative indices.
~730, ~720	ρ_r(CH_2_) (orthorhombic)	Doublet preserved; no systematic loss—consistent with modest crystallinity changes.
1700–1740	C=O (carbonyl)	Not detected; Carbonyl Index effectively ~0 within noise.
910–990	=CH_2_/vinyl	Not evident; no unsaturation growth.

## Data Availability

The original contributions presented in this study are included in the article. Further inquiries can be directed to the corresponding author.
